# Supplementary material for: Alcohol outlet density and adolescent drinking behaviors in Thailand, 2007–2017: A spatiotemporal mixed model analysis
Source: PLoS One. 2024 Oct 31;19(10):e0308184. doi: 10.1371/journal.pone.0308184 (PMC11527161; doi:10.1371/journal.pone.0308184)
Supplement: Attachment — Submitted filename: Response to Reviewers.docx [file pone.0308184.s001.docx]

**PONE-D-23-42353**

**Response to Reviewers**

Dear Dr. Mendes,

Thank you for giving us the opportunity to submit a revised draft of the manuscript “Alcohol outlet density and adolescent drinking behaviors in Thailand, 2007–2017: A spatiotemporal mixed model analysis” for publication in *PLOS ONE*. We appreciate the time and effort that you and the reviewers dedicated to providing feedback on our manuscript and are grateful for the insightful comments on and valuable improvements to our paper. We have incorporated most of the suggestions provided by the reviewers. These changes are marked-up within the manuscript using tracked changes. Please see the blue part below for a point-by-point response to the reviewers’ comments and concerns. All page numbers refer to the revised manuscript file with tracked changes.

**Journal Requirements**:

1. When submitting your revision, we need you to address these additional requirements.

Please ensure that your manuscript meets PLOS ONE's style requirements, including those for file naming. The PLOS ONE style templates can be found at

<https://journals.plos.org/plosone/s/file?id=wjVg/PLOSOne_formatting_sample_main_body.pdf> and https://journals.plos.org/plosone/s/file?id=ba62/PLOSOne_formatting_sample_title_authors_affiliations.pdf

Author response: Thank you for your comment. We have checked that our manuscript meets the target journal’s requirements.

2. In the online submission form, you indicated that The data underlying the results presented in this study are available upon request from the corresponding author.

All PLOS journals now require all data underlying the findings described in their manuscript to be freely available to other researchers, either a. In a public repository, b. Within the manuscript itself, or c. Uploaded as supplementary information.

This policy applies to all data except where public deposition would breach compliance with the protocol approved by your research ethics board. If your data cannot be made publicly available for ethical or legal reasons (e.g., public availability would compromise patient privacy), please explain your reasons on resubmission and your exemption request will be escalated for approval.

Author response: Due to ethical limitations governing the dissemination of a de-identified dataset, it is not permissible to share the data publicly. We assert that the manuscript in its present form includes all necessary information pertinent to our study. Researchers who fulfill the criteria for accessing confidential data are urged to contact the corresponding author to facilitate discussions regarding their requests for data access.

3. Please include your full ethics statement in the ‘Methods’ section of your manuscript file. In your statement, please include the full name of the IRB or ethics committee who approved or waived your study, as well as whether or not you

obtained informed written or verbal consent. If consent was waived for your study, please include this information in your statement as well.

Author response: Thank you for your comment. We have incorporated the ethics statement in the Methods section accordingly.

The revised text reads as follows in the “Study design” section from lines 122-125:

*“The authors have applied for an amendment regarding protocols for analyses of anonymized secondary data. All obtained data were anonymous. Ethical approval was obtained from the Human Research Ethics Committee of the Faculty of Medicine, Prince of Songkla University (REC. 62-054-18-1).”*

4. We note that Figure 2 in your submission contain map images which may be copyrighted. All PLOS content is published under the Creative Commons Attribution License (CC BY 4.0), which means that the manuscript, images, and Supporting Information files will be freely available online, and any third party is permitted to access, download, copy, distribute, and use these materials in any way, even commercially, with proper attribution.

For these reasons, we cannot publish previously copyrighted maps or satellite images created using proprietary data, such as Google software (Google Maps, Street View, and Earth). For more information, see our copyright guidelines: http://journals.plos.org/plosone/s/licenses-and-copyright.

We require you to either present written permission from the copyright holder to publish these figures specifically under the CC BY 4.0 license, or remove the figures from your submission:

a. You may seek permission from the original copyright holder of Figure 2 to publish the content specifically under the CC BY 4.0 license.

We recommend that you contact the original copyright holder with the Content Permission Form (http://journals.plos.org/plosone/s/file?id=7c09/content-permission-form.pdf) and the following text:

“I request permission for the open-access journal PLOS ONE to publish XXX under the Creative Commons Attribution License (CCAL) CC BY 4.0 (http://creativecommons.org/licenses/by/4.0/). Please be aware that this license allows unrestricted use and distribution, even commercially, by third parties. Please reply and provide explicit written permission to publish XXX under a CC BY license and complete the attached form.”

Please upload the completed Content Permission Form or other proof of granted permissions as an "Other" file with your submission.

In the figure caption of the copyrighted figure, please include the following text: “Reprinted from (Drefs) under a CC BY license, with permission from [name of publisher], original copyright [original copyright year].”

b. If you are unable to obtain permission from the original copyright holder to publish these figures under the CC BY 4.0 license or if the copyright holder’s requirements are incompatible with the CC BY 4.0 license, please either i) remove the figure or ii) supply a replacement figure that complies with the CC BY 4.0 license. Please check copyright information on all replacement figures and update the figure caption with source information. If applicable, please specify in the figure caption text when a figure is similar but not identical to the original image and is therefore for illustrative purposes only.

The following resources for replacing copyrighted map figures may be helpful:

USGS National Map Viewer (public domain): http://viewer.nationalmap.gov/viewer/
The Gateway to Astronaut Photography of Earth (public domain): http://eol.jsc.nasa.gov/sseop/clickmap/
Maps at the CIA (public domain): https://www.cia.gov/library/publications/the-world-factbook/index.html and https://www.cia.gov/library/publications/cia-maps-publications/index.html
NASA Earth Observatory (public domain): http://earthobservatory.nasa.gov/
Landsat: http://landsat.visibleearth.nasa.gov/
USGS EROS (Earth Resources Observatory and Science (EROS) Center) (public domain): http://eros.usgs.gov/# Natural Earth (public domain): http://www.naturalearthdata.com/

Author response: Thank you for pointing this out. The map in Figure 2 was created using QGIS, Open Source Geographic Information System (GIS) licensed under the GNU General Public License. Hence, we believe that we hold the copyright to the maps. Additionally, we have revised a sentence in the Statistical analysis section to give a clearer explanation of this topic.

The revised text reads as follows in the “Statistical analysis” section from lines 210-211:

*“We created the map using “QGIS,” an open source Geographic Information System (GIS).”*

If you have any further questions or require clarification on this matter, please do not hesitate to let us know.

5. Please include captions for your Supporting Information files at the end of your manuscript, and update any in-text citations to match accordingly. Please see our Supporting Information guidelines for more information:

http://journals.plos.org/plosone/s/supporting-information.

Additional Editor Comments:

Author response: There is no supporting information file due to the reason mentioned above.

Dear Authors,
The reviewers have indicated a number of changes and suggestions in the manuscript and in the current format it cannot be considered for publication. I suggest revising the manuscript and submitting the revised version for a new analysis.
Sincerely, Larissa

[Note: HTML markup is below. Please do not edit.]

Reviewers' comments:
Reviewer's Responses to Questions

**Comments to the Author**

1. Is the manuscript technically sound, and do the data support the conclusions?

The manuscript must describe a technically sound piece of scientific research with data that supports the conclusions. Experiments must have been conducted rigorously, with appropriate controls, replication, and sample sizes. The conclusions must be drawn appropriately based on the data presented.

Reviewer #1: Yes Reviewer #2: Yes

2. Has the statistical analysis been performed appropriately and rigorously?

Reviewer #1: Yes Reviewer #2: Yes

3. Have the authors made all data underlying the findings in their manuscript fully available?

The PLOS Data policy requires authors to make all data underlying the findings described in their manuscript fully available without restriction, with rare exception (please refer to the Data Availability Statement in the manuscript PDF file). The data should be provided as part of the manuscript or its supporting information, or deposited to a public repository. For example, in addition to summary statistics, the data points behind means, medians and variance measures should be available. If there are restrictions on publicly sharing data—e.g. participant privacy or use of data from a third party—those must be specified.

Reviewer #1: No Reviewer #2: Yes

4. Is the manuscript presented in an intelligible fashion and written in standard English?

PLOS ONE does not copyedit accepted manuscripts, so the language in submitted articles must be clear, correct, and unambiguous. Any typographical or grammatical errors should be corrected at revision, so please note any specific errors here.

Reviewer #1: Yes Reviewer #2: Yes

5. Review Comments to the Author

Please use the space provided to explain your answers to the questions above. You may also include additional comments for the author, including concerns about dual publication, research ethics, or publication ethics. (Please upload your review as an attachment if it exceeds 20,000 characters)

**Reviewer #1**: The study's aim was to explore the relationship between alcohol outlet density and drinking behaviors among Thai adolescents. The manuscript is well-written and has interesting methodology and results, especially to base actions to prevent and combat alcohol consumption by adolescents. However, some minor details need to be revised, mainly in the introduction, and the conclusion with the implications needs to be improved and present more advanced discussions on public policies.

Author response: Thank you for your valuable suggestion. We agree with your comment and have incorporated your suggestions throughout the manuscript.

**Introduction**

The introduction needs a revision of the information presented; readers from outside Thailand need to understand everything only by reading your manuscript.

Author response: Thank you for your valuable suggestion. Accordingly, throughout the introduction, we have included additional information regarding Thailand’s alcohol control laws and the current situation of alcohol consumption in Thailand, as well as modified some parts of the introduction to facilitate better logic.

The revised text reads as follows in the “Introduction” section from lines 27-28:

*“Alcohol is a major threat to the individual and society as a whole. The effects on individual health range from cardiovascular diseases and infectious diseases to injuries. It can lead to suicide and interpersonal violence.”*

*lines 53-66:*

*“The World Health Organization has introduced SAFER strategies as alcohol control interventions to reduce harmful alcohol use. The strategies consist of the following: (1) Strengthen restriction on alcohol availability; (2) Advance and enforce drunk driving countermeasures; (3) Facilitate access to screening, brief interventions, and treatment; (4) Enforce bans or comprehensive restrictions on alcohol advertising, sponsorship, and promotion; and (5) Raise prices on alcohol through excise taxes and pricing policies.*

*Currently, Thailand has three major national laws that are in accordance with the SAFER strategies: the Alcoholic Beverage Control Act B.E. 2551 (2008), Excise Act B.E. 2560 (2017), and Road Traffic Act B.E. 2522 (1979) [11]. The Alcoholic Beverage Control Act B.E. 2551 (2008) is Thailand's first legislative effort aimed specifically at controlling the consumption and distribution of alcoholic beverages. The Excise Act B.E. 2560 (2017) regulates the taxation and licensing of production, and the sales and import of products and services, including alcoholic products. In the context of alcohol regulation, the Road Traffic Act B.E.2522 (1979) sets limits on the alcohol blood content (BAC) for drivers.”*

*lines 71-72:*

*“Moreover, a study has shown that Thai adolescents are inclined to start drinking earlier than in the past.”*

And I recommend including the hypothesis of your study after the objective.

Author response: Thank you for this suggestion. We agree with your comment. Therefore, we have included the hypothesis as follows on lines 104-105:

*“Our hypothesis was as follows: the higher the alcohol density, the higher the proportion of adolescents’ alcohol use.”*

We have also incorporated the interpretation of our results based on the hypothesis in the “Discussion” section on lines 271-272.

*“This result was consistent with our hypothesis that the higher the density, the more adolescents would drink.”*

Line 2-3: Are these efforts just in online settings?

Author response: Thank you for pointing this out. We have revised this sentence to provide more insight into the marketing efforts of the alcohol industry and their effects.

The revised text reads as follows on lines 50-52:

*“To exacerbate the situation, there has been an increase in various marketing efforts from the alcohol industry, including mass media commercials, in-store displays, merchandise, and online marketing.”*

Line 3-4: We do not know this Alcoholic Beverage Control Act B.E. 2551. What is it? A Thailand national law?

Author response: Thank you for pointing this out. We have included additional information about the Alcoholic Beverage Act B.E. 2551.

The revised text reads as follows on lines 59-63:

*“Currently, Thailand has three major national laws that are in accordance with the SAFER strategies: the Alcoholic Beverage Control Act B.E. 2551 (2008), Excise Act B.E. 2560 (2017), and Road Traffic Act B.E. 2522 (1979). The Alcoholic Beverage Control Act B.E. 2551 (2008) is Thailand's first legislative effort aimed specifically at controlling the consumption and distribution of alcoholic beverages. …”*

Line 18: We do not know this Excise Act B.E. 2560. What is it?

Author response: Thank you for pointing this out. We have included additional information about the Excise Act B.E. 2560.

The revised text reads as follows on lines 59-64:

*“…Currently, Thailand has three major national laws that are in accordance with the SAFER strategies: the Alcoholic Beverage Control Act B.E. 2551 (2008), Excise Act B.E. 2560 (2017) and Road Traffic Act B.E. 2522 (1979). …. The Excise Act B.E. 2560 (2017) regulates the taxation and licensing of production, and the sales and import of products and services, including alcoholic products. …”*

Line 20-23: Is this information important here? (about the types of permits)

Author response: Thank you for pointing this out. We originally included this information to provide background on the type of sale permits, which would be mentioned later in the strengths and limitations section. However, upon review, we have determined that this information is not necessary here. Thus, we have removed it.

Line 24: Which campus?

Author response: Thank you for pointing this out. Details regarding “campus” from the literature have been added on line 87.

*“A study in New Zealand examined the relationship between alcohol consumption and licenses granted within a 3 km radius of six university campuses and found that alcohol license density was positively correlated with drinkers and alcohol-related problems as well as alcohol consumption among adolescents.”*

Line 27-37: I recommend reading again and adjusting the order in which the information appears. I could not understand much of your purpose with this paragraph; it is confusing.

Author response: We agree with the reviewer’s assessment. Accordingly, throughout the paragraph, we have made revisions by removing unnecessary sentences, reordering the information, incorporating additional evidence, and rephrasing some sentences as follows on lines 90-107:

*“Although a growing body of studies has documented the relationship between alcohol license density and alcohol consumption behaviors in adolescents, there remains a marked gap in the literature regarding the effects in the context of developing countries. The existing studies were predominantly single-centre studies conducted mostly in developed countries [28, 29]. A critical review, focusing primarily on literature from developed countries, highlighted the need for diverse research settings as different jurisdictions have varying norms and legislation [30]. Furthermore, much of the existing research in developing countries has been limited due to the methodological constraints. For instance, they are mostly based on single-year data [31-33]. Moreover, most studies have used longitudinal or panel data to track temporal changes [34], with outcomes including intimate partner violence [35-38], drunk driving [39], motorcycle accidents [40], and hospitalization rates [34], rather than focusing directly on alcohol consumption as the outcome.*

*To address this gap in knowledge, we aimed to examine the relationship between alcohol license density and the proportion of alcohol consumption among adolescents at the provincial level from 2007 to 2017 in Thailand. Our hypothesis was as follows: the higher the alcohol density, the higher the proportion of adolescents’ alcohol use. Our findings will help bridge the gap between existing research in developed and developing countries context as well as informs policymakers of measures for controlling liquor licenses in Thailand.”*

**Materials and Methods**

I recommend you analyze if you can say your study has a temporal analysis.

Author response: Thank you for your suggestion. The terms have been corrected in the “Statistical analysis” section on lines 190-192.

The revised text reads as follows:

“*This model allowed us to analyze how the temporal dynamics—reflected through the survey years—impact alcohol consumption patterns across different provinces, alongside the spatial distribution of alcohol sales licenses.”*

Line 44: Your study is an analysis of secondary data, not a secondary analysis.

Author response: Thank you for pointing this out. The terms have been corrected on lines 111-112

The revised text reads as follows:

“*This was a cross-sectional study involving an analysis of secondary data: nationally representative surveys and pre-existing sales license data.”*

Line 45-46: Include information about the data of alcohol establishments.

Author response: Thank you for your suggestion. Upon revision, we have found that the order of the information related to outlets and consumption can cause confusion. Hence, we have revised the Study design and Data collection sections by adjusting the order of information and incorporating the suggested information about the data of alcohol establishments.

In the “Study design” section, the revised text reads as follows on lines 111-121:

“*This was a cross-sectional study involving an analysis of secondary data: nationally representative surveys and pre-existing sales license data. For the alcohol consumption data, we utilized data from the 2007, 2011, and 2017 waves of the Tobacco and Alcohol Consumption Surveys. They are nation-wide alcohol consumption surveys that have been conducted every four years by Thailand’s National Statistical Office since 2001.*

*For the alcohol outlet data, we employed the aggregate data on the number of alcohol sales licenses, involving the total number of licenses approved by Thailand’s Excise Department, Ministry of Finance, for each province in the years 2007, 2011 and 2017. The dataset also included the annual statistical information report on the population and households from the official statistics registration systems of the Department of Provincial Administration, Ministry of the Interior, Thailand.*”

In the “Data collection” section, the revised text reads as follows on lines 134-143:

*“We obtained the secondary data concerning alcohol consumption and alcohol sales licenses directly from the Thai National Statistical Office and the Excise Department, respectively. Originally, all alcohol consumption survey waves employed a two-stage stratified sampling approach. The provinces were chosen as strata. The units for the first and second stages were villages and households, respectively. The villages were chosen in proportion to the population of each province. The households were systematically selected. However, notably, despite these methodological differences, the survey population and data collection procedures remained consistent across all three waves. In each instance, data were collected through face-to-face interviews, encompassing individuals aged 15 years and older from all geographical regions, provinces, and districts within Thailand. All data were accessed online on January 11, 2021.”*

Line 65: You need to make it clear that you collected information on establishments from 2007 to 2018, and that all the years have information. I only understood this in the results.

Author response: Thank you for pointing this out. We have included the suggested information in the “Study design” section

The revised text reads as follows on lines 116-118:

“*For the alcohol outlet data, we employed the aggregate data on the number of alcohol sales licenses, involving the total number of licenses approved by Thailand’s Excise Department, Ministry of Finance, for each province in the years 2007, 2011 and 2017.*”

Reading the methodology, I had a question: was the density of establishments selling alcohol estimated for each province, and to associate it with consumption by teenagers, was this density analyzed by province? Or was it by the area around the houses?

Author response: Thank you for the question. The density was estimated for each province. We also analyzed the association between density and consumption using provincial data. Additionally, we have revised the paragraph in the Statistical analysis section to give a clearer explanation of this topic.

The revised text reads as follows on lines 204-205:

“*We applied a heatmap to visualize the distribution of the dependent (density of alcohol sales licenses in each province) and independent variables (proportion of alcohol consumption).*”

Line 130-132: You must include all the variables analyzed. The readers do not go to table 1 to discover all the variables you include in the analysis.

Author response: Thank you for your suggestion. Upon revision , we have found that the confounding variable should be explained in the Potential confounding variable: social deprivation index and other demographics section, which lacked clarity. Hence, we have revised the sentence by rephrasing and arranging the order in the confounding section as follows on lines 174-176:

“*The potential confounding variables include the social deprivation index, survey year, region of residence, household area, number of household members, marital status, educational level, monthly household income, and smoking status.”*

And on line 183:

*“All confounding variables were collected and analyzed for each participant.”*

Additionally, we have removed the following sentence from the Statistical analysis section to avoid confusion:

“*Examples include sex, education, health-risk behaviors (e.g., smoking or substance abuse),  socioeconomic status, and family factors.*”

**Results**

Figure 1: Include the location in the title of the figure.

Author response: As suggested by the reviewer, we have included the location of the study in the figure’s title. The revised title now reads:

“***Fig 1. Number of Liquor Licenses per 1,000 Inhabitants in 2007–2018 in Thailand.***”

Table 2: Include the total number of participants of your study in the title of the table.

Author response: As suggested by the reviewer, we have included the total number of participants in the figure’s title. The revised title now reads:

**“*Table 2. Association of Density of Alcohol Sales Licenses, Survey Year, and Social Deprivation Index with Proportion of Alcohol Consumption among Thai Adolescents: Results From a Spatiotemporal Mixed Model Analysis*. *(N=9,566)*”**

Figure 2: Review the figure to see some mistakes, and I recommend writing a note explaining that the scales vary through the categories.

Author response: Thank you for your suggestion. We have revised the figure and its title by clarifying the terms, correcting the fonts, and including the note relating to the scales. The revised title now reads:

**“*Figure 2. Map of the Density of Alcohol Sales Licenses and Proportion of Alcohol Consumption among Thai Adolescents in 2007, 2011, and 2017*.”**

**Discussion**

Line 235-243: What is your hypothesis as to why these episodes of heavy drinking are more frequent among female adolescents?

Author response: Thank you for pointing this out. We have included our hypothesis in the discussion accordingly.

The revised text reads as follows on line 312-318:

*“We found an increased incidence of heavy episodic drinking in female adolescents. Considering that the vigorous marketing efforts targeting women and the modern Western lifestyle, characterized by shifted gender roles due to higher education and economic empowerment, influenced young Thai women to engage in heavy episodic drinking, we proposed that the abundant availability of alcohol could further encourage consumption. Nonetheless, our result contradicts data from Korea, where a decrease in alcohol consumption in the same group was observed.”*

And on line 324-328:

*“It should be noted that the increased consumption in Thai female adolescents is concerning as an early onset of alcohol use correlates with a higher likelihood of heavy episodic drinking among Thai females. Those who begin drinking before the age of 20 show greater odds of engaging in heavy episodic drinking compared to those who start at 25 or older. This relationship appears stronger among females than males.”*

**Conclusion**

Have you only thought about regulating licenses and limiting teenagers' consumption? I recommend a deeper and more advanced analysis of the issue. Wouldn't education actions with teenagers be necessary to raise awareness about alcohol consumption and its harms? Just limiting or prohibiting alcohol is not working, as this is already happening, and the results of your study show that teenagers still consume alcohol. I recommend reviewing the implications and suggestions (and changing the abstract as well).

Author response: Thank you for your suggestion. We have included other alcohol control initiatives in the conclusion accordingly.

The revised text reads as follows on lines 373-377:

*“Moreover, Thailand can reduce adolescent alcohol use by strengthening the SAFER strategies: enforcing stricter sales and age restrictions, enhancing drink-driving enforcement, expanding youth-targeted treatments, banning appealing alcohol ads to youths, and raising alcohol prices. These steps will make alcohol less accessible and appealing to young people.”*

**Reviewer #2:** At the beginning of the abstract the authors say: “This study aims to explore the relationship between alcohol outlet density and drinking behaviors among Thai adolescents” and at the end of the introduction they say: “This study examines the relationship between alcohol outlet density and the proportion of alcohol use among Thai teenagers.” Review the end of the introduction and standardize for consumer behavior.

Author response: Thank you for pointing this out. The reviewer is correct, and we have standardized the terms in the abstract and introduction sections accordingly.

In the “abstract”, the revised text reads as follows:

“*This study aimed* *to explore the relationship between alcohol outlet density and the proportion of alcohol consumption among Thai adolescents.*”

In the “introduction”, the revised text reads as follows from line 102-104:

“*To address this gap in knowledge, we aimed to examine the relationship between alcohol license density and the proportion of alcohol consumption among adolescents at the provincial level from 2007 to 2017 in Thailand.”*

In the methodology (line 72) it mentions that they are drinking behaviors, and it is important to change the term “the proportion” at the end of the introduction

Author response: We agree with the reviewer’s assessment. Accordingly, throughout the manuscript, we have standardized the outcome variable to proportion of alcohol consumption. The changes have been made in the Dependent variable section and thoughout the manuscript.

In the dependent variable section, the revised text reads as follows from line 144-161:

***“Dependent variable: Proportion of alcohol consumption***

*The outcome variable was proportion of alcohol consumption, including the proportion of current drinkers, regular drinkers, and heavy episodic drinkers (HEDs). To categorize the drinkers, we collected drinking frequency and the quantity of alcohol consumed as variables. The proportion was calculated as follows:*

- *We calculated the proportion of* ***current drinkers*** *as the number of participants who drank at least one standard drink of alcohol during the 12 months preceding the interview and divided by the number of all participants.*
- *We calculated the proportion of* ***regular drinkers*** *using the number of current drinkers who drank alcohol at least once per week or more, divided by the number of all current drinkers. Regular drinking is related to the lifetime risk of hospitalization for alcohol-related problems [41, 42].*
- *We calculated the proportion of* ***HEDs*** *by dividing the number of current drinkers—those who drank at least four to five standard alcoholic drinks on at least one occasion—by the number of all current drinkers. This is one of the most important indicators of acute alcohol-related harm (e.g., injuries, accidents, and acute social consequences) [43] and chronic diseases (e.g., tuberculosis, epilepsy, ischemic heart disease, and cirrhosis) [44]. These can be provided by alcohol intoxication and drinking intensity [45].”*

The study is confusing in determining a target audience, in the title and objective they mention that they are teenagers, but in the introduction (line 30) they mention young adults. It is important to define the age range of the group studied and standardize the terms

Author response: Thank you for pointing this out. We agree with this comment. Therefore, we have standardized the terms to adolescents, which best fit with our sample population aged 15-19 years old, throughout the manuscript.

Line 106 -107: As potential confounding variables for each participant, some variables were included that I don't think apply to the lives of adolescents (study participants), such as marital status and monthly income. That's why it's important to make it clear who is being studied. If they are adults (which I don't believe they are) these adjustment variables are relevant. Other interesting adjustment variables for adolescents, for example: age, occurrence of bullying, difficulty interacting with peers, satisfaction with school life, alcohol consumption by someone in the family

Author response: Thank you very much. You have raised an important point here. As our sample population is aged 15-19 years old, some values of marital status, educational level, and monthly household income variables may sound irrelevant. Please let us explain the variables point by point. First, for marital status, under the Thai law, an individual can marry at the age of 17 years old with parental consent. Thus, we think that married/divorced statuses are possible. Second, for monthly household income, from the term used in the original survey, the participants might have answered their parents as contributing to the household income. Some might have their own income. Third, for education level, we assume that the term used in the original survey might be confusing; thus, the participants might answer the education they received at the time of the survey. In conclusion, considering the reasons above, we think that the variables are plausible for our sample population.

Thank you for your recommendation regarding the adjustment variables. Although we agree that this is an important consideration, it is beyond the scope of this manuscript because these variables are not included in our objective; we utilized secondary data that did not include these variables.

Line 115: The confounding variables described can be found in lines 106, 107 and 108.

Author response: Thank you for pointing this out. We have revised the sentence originally in lines 106-108 by rephrasing and arranging the order in the confounding section for clarity as follows on lines 174-176:

“*The potential confounding variables include social deprivation index, survey year, region of residence, household area, number of household members, marital status, educational level, monthly household income, and smoking status.”*

Additionally, we have removed the following sentence from Statistical analysis to avoid confusion:

“*Examples include sex, education, health-risk behaviors (e.g., smoking or substance abuse),  socioeconomic status, and family factors.*”

In the introduction, an approach to the impacts of alcohol consumption on the health of adolescents/young adults would be interesting.

Author response: Thank you for pointing this out. We agree with this comment. Therefore, we have included the health outcomes of alcohol in adolescents in the introduction.

The revised text reads as follows on lines 48-50

“*Specifically, alcohol consumption in adolescents can lead to cognitive function deficit [3], an increased risk of psychological distress [4], development of severe liver disease [5], and alcohol dependence in adulthood [6].”*

6. PLOS authors have the option to publish the peer review history of their article (what does this mean?). If published, this will include your full peer review and any attached files.

If you choose “no”, your identity will remain anonymous but your review may still be made public.

**Do you want your identity to be public for this peer review?** For information about this choice, including consent withdrawal, please see our Privacy Policy.

Reviewer #1: Yes: Luana Lara Rocha Reviewer #2: No

If reviewer comments were submitted as an attachment file, they will be attached to this email and accessible via the submission site. Please log into your account, locate the manuscript record, and check for the action link "View Attachments". If this link does not appear, there are no attachment files.

While revising your submission, please upload your figure files to the Preflight Analysis and Conversion Engine (PACE) digital diagnostic tool, https://pacev2.apexcovantage.com/. PACE helps ensure that figures meet PLOS requirements. To use PACE, you must first register as a user. Registration is free. Then, login and navigate to the UPLOAD tab, where you will find detailed instructions on how to use the tool. If you encounter any issues or have any questions when using PACE, please email PLOS at figures@plos.org. Please note that Supporting Information files do not need this step.

Author response: Thank you. We have used PACE for all of our figures.
